# Search for copy number variants in chromosomes 15q11-q13 and 22q11.2 in obsessive compulsive disorder

**DOI:** 10.1186/1471-2350-11-100

**Published:** 2010-06-21

**Authors:** Richard Delorme, Daniel Moreno-De-Luca, Aurélie Gennetier, Wolfgang Maier, Pauline Chaste, Rainald Mössner, Hans Jörgen Grabe, Stephan Ruhrmann, Peter Falkai, Marie-Christine Mouren, Marion Leboyer, Michael Wagner, Catalina Betancur

**Affiliations:** 1 INSERM, U955, Institut Mondor de Recherche Biomédicale, Psychiatric Genetics, Créteil, France; 2 AP-HP, Hôpital Robert Debré, Department of Child and Adolescent Psychiatry, Paris, France; 3 INSERM, U952, Paris, France; 4 CNRS, UMR 7224, Paris, France; 5 UPMC Univ Paris 06, Paris, France; 6 Department of Psychiatry and Psychotherapy, University of Bonn, Bonn, Germany; 7 Department of Psychiatry and Psychotherapy, University of Greifswald, Stralsund, Germany; 8 Department of Psychiatry and Psychotherapy, University of Cologne, Cologne, Germany; 9 Department of Psychiatry and Psychotherapy, University of Göttingen, Göttingen, Germany; 10 AP-HP, Henri Mondor-Albert Chenevier Hospital, Department of Psychiatry, Créteil, France; 11 Université Paris 12, Faculty of Medicine, Créteil, France

## Abstract

**Background:**

Obsessive-compulsive disorder (OCD) is a clinically and etiologically heterogeneous syndrome. The high frequency of obsessive-compulsive symptoms reported in subjects with the 22q11.2 deletion syndrome (DiGeorge/velocardiofacial syndrome) or Prader-Willi syndrome (15q11-13 deletion of the paternally derived chromosome), suggests that gene dosage effects in these chromosomal regions could increase risk for OCD. Therefore, the aim of this study was to search for microrearrangements in these two regions in OCD patients.

**Methods:**

We screened the 15q11-13 and 22q11.2 chromosomal regions for genomic imbalances in 236 patients with OCD using multiplex ligation-dependent probe amplification (MLPA).

**Results:**

No deletions or duplications involving 15q11-13 or 22q11.2 were identified in our patients.

**Conclusions:**

Our results suggest that deletions/duplications of chromosomes 15q11-13 and 22q11.2 are rare in OCD. Despite the negative findings in these two regions, the search for copy number variants in OCD using genome-wide array-based methods is a highly promising approach to identify genes of etiologic importance in the development of OCD.

## Background

Obsessive-compulsive disorder (OCD) is characterized by recurrent and intrusive thoughts and ritualistic behaviors or mental acts that a person feels compelled to perform. Although the etiology of OCD remains unknown, the results of twin studies, familial studies, and segregation analyses have provided compelling evidence that OCD has a strong genetic component [1]. However, OCD fails to follow Mendelian patterns of inheritance and is considered a complex genetic disorder. Several theoretically relevant functional candidate genes have been examined in OCD, but no susceptibility genes have yet been identified with certainty [1]. Like in other neuropsychiatric conditions, the difficulty in identifying the responsible genes may be the consequence of the clinical and genetic heterogeneity of the disorder.

**Figure 1:**
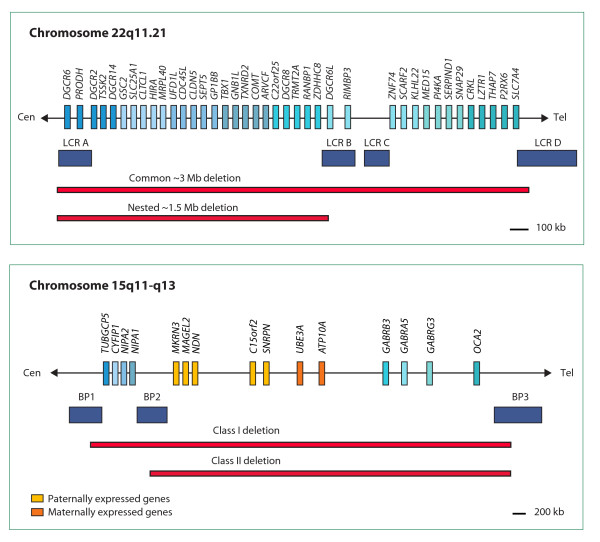
**22q11.21 and 15q11-q13 regions**. **A**. Schematic representation of chromosome 22q11.21 showing the genes in the region, the common recurrent deletions (red bars), and the segmental duplications that mediate the rearrangements (blue rectangles), termed low copy repeat (LCR) A to D. **B**. Schematic representation of the 15q11-q13 region deleted in Prader-Willi and Angelman syndromes. Paternally expressed genes are shown in yellow, maternally expressed genes in orange. Recurrent deletions are represented as red bars; the segmental duplications that mediate the rearrangements, termed breakpoint (BP) 1 to 3, are portrayed as blue rectangles.

Chromosomal rearrangements have been reported in a small number of individuals with OCD [2-7]. Two microdeletion disorders, 22q11.2 deletion syndrome and Prader-Willi syndrome (PWS), due to deletion of chromosome 15q11-13, are frequently associated with obsessive-compulsive symptoms, suggesting that gene dosage effects at these two loci could contribute to the development of the obsessive-compulsive phenotype. 22q11.2 deletion syndrome, also known as DiGeorge or velocardiofacial syndrome, is a highly variable disorder caused by a microdeletion of chromosome 22q11.2, often occurring de novo (80%), with an estimated prevalence of 1/2000-6000 live births [8,9]. The majority of 22q11.2 deletions (87%) are ~3 Mb in size, whereas a small proportion (8%) involves smaller nested ~1.5 Mb deletions [10] (Figure 1). 22q11 deletion syndrome is characterized by cardiac malformations, immunodeficiency, dysmorphic features, and palate anomalies, associated with cognitive impairment. Comorbid psychophathologies are frequent, and include schizophrenia, attention deficit-hyperactivity disorder, autism spectrum disorders and OCD or obsessive-compulsive symptoms [11]. In particular, 33% (14/43) of 22q11.2 deletion subjects meet criteria for OCD and an even higher proportion report obsessive-compulsive symptoms (83%) [12], suggesting that the 22q11.2 locus harbors genes that could predispose to OCD. Other researchers have also noted increased rates of marked obsessive-compulsive symptoms and OCD among patients with 22q11 deletion syndrome, with rates of OCD of 8% (2/25) [13], 10.7% (3/28) [14], and 14% (2/14) [15]. In contrast to the study by Gothelf and colleagues [12], the latter studies did not search systematically for OCD in their patients, which might explain the lower frequency. The *COMT *gene, coding for the enzyme catechol-O-methyltransferase, responsible for dopamine and norepinephrine inactivation, is one of the prominent candidate genes for susceptibility to mental disorders located in this interval, and is considered a major candidate gene in OCD [16].

PWS is the result of the loss of expression of several imprinted genes located in the 15q11-q13 region, which are normally expressed on the paternally derived chromosome [17] (Figure 1). In 70% of PWS patients, a paternal 15q11-q13 deletion is found. The remaining have a uniparental maternal disomy 15 (~25%) or an imprinting defect (~5%). This neurodevelopmental disorder has an estimated prevalence of 1/10000 live births and is characterized by infantile hypotonia, neonatal feeding difficulties, hypogonadism, hyperphagia (leading to obesity in early childhood) and cognitive deficits. Many studies have also reported a range of obsessive-compulsive and ritualistic behaviors not related to food in about 50% of PWS patients, including skin picking, hoarding, concerns with symmetry, exactness, ordering and arranging, need to tell or ask, and insistence on routines [18-21]. Thus, paternally expressed genes within the PWS critical region (e.g., *MKRN3*, *MAGEL2*, *NDN*, and *SNRPN-SNURF*) could represent risk factors for OCD.

The aim of our study was to search in a sample of OCD patients for the presence of 15q11-13 or 22q11.2 microrearrangements using multiplex ligation-dependent probe amplification (MLPA). This is the first study to systematically screen these two copy number variants (CNVs) in OCD patients. MLPA has the ability to analyze up to 50 targets in a single reaction and to detect both deletions and duplications. The efficacy of MLPA in detecting 15q11-q13 and 22q11.2 microrearrangements has been previously established [22,23]. We thus applied MLPA to screen our dataset for 15q11-q13 and 22q11.2 deletions/duplications.

## Methods

### Subjects

The study included 236 probands seeking treatment at university-based OCD clinics in Paris and in Germany (Table 1). Patients had to meet DSM-IV criteria for full OCD [24], had to have OCD as their main disorder, and had to be of European descent to be included in the study. Patients with clear dysmorphic features or severe mental retardation were excluded from the study. However, no neuropsychological tests were performed to determine the intellectual quotient of patients enrolled in the study. Lifetime psychiatric evaluation was carried out during a direct interview by trained psychiatrists using either the French or German version of the Diagnostic Interview for Genetic Studies (DIGS) [25] for patients over 17 years of age, or the Kiddie Schedule for Affective Disorders and Schizophrenia - Epidemiologic version (K-SADS-E) [26] for probands under 17 years of age. Among the French probands, 31% (22/71) had a history of chronic tic disorders (including chronic motor or vocal tics and Tourette syndrome), 23% (16/71) had a family history of OCD and 11% (8/71) had a family history of chronic tic disorders. No equivalent data was available for the German subjects. The local Research Ethics Boards approved the study protocol. Written informed consent was obtained from all participating subjects. If the proband was under 18 years old, the proband's consent and written parental consent were obtained.

**Table 1:** Clinical and demographic characteristics of OCD probands

	French probands (n = 71)	German probands (n = 165)	All (n = 236)
Male/female	44/27	79/86	123/113
Age at interview (yrs)	22.2 ± 14.3	36.4 ± 12.9	31.7 ± 15.0
Age at onset of OCD (yrs)	12.7 ± 8.8	19.3 ± 10.8	17.3 ± 10.6
Y-BOCS total score^a^	28.3 ± 5.4	17.2 ± 9.5	17.9 ± 9.1

^a ^Y-BOCS, Yale-Brown Obsessive Compulsive Scale. Values are expressed as mean ± SD.

### MLPA

Genomic DNA was extracted from peripheral blood leukocytes or lymphoblastoid cell lines using the NucleoSpin Blood L kit (Macherey-Nagel, Duren, Germany). MLPA was performed using the P064 MR1 (mental retardation 1) and P250 DiGeorge kits (MRC-Holland, Amsterdam, The Netherlands). The P064 MR1 kit detects copy number changes both at the 15q and 22q loci, and includes 5 probes specific for sequences in or near the 15q11.2 Prader-Willi syndrome/Angelman syndrome critical region (one probe in the *MKRN3*, *NDN*, and *GABRB3*, and two in *UBE3A*) and 6 probes in the 22q11.21 DiGeorge region (in chromosomal order: *CLTCL1, CDC45L, CLDN5, ARVCF, KLHL22, SNAP29*). Information regarding the probe sequences and ligation sites can be obtained at http://www.mlpa.com. The P064 kit also screens for other mental retardation syndromes: Smith-Magenis syndrome (17p11.2), Williams syndrome (7q11.23), 1p deletion syndrome (1p36), Sotos syndrome (5q35.3), Miller-Dieker syndrome (17p13.3), Alagille syndrome (20p12.2), and Saethre-Chotzen syndrome (7p21). All patients (n = 236) were screened with the P064 MLPA kit; in addition, 126 patients were also screened with the P250 DiGeorge kit, which contains 14 different probes in the 22q11.2 region.

Fifty nanograms of DNA were used in the MLPA protocol. Experiments were performed according to the manufacturer's instructions but the volume of all kit reagents was decreased by 20%. Reactions were performed on a GeneAmp PCR System 9700 (Applied Biosystems, Foster City, CA, USA). PCR products were analyzed by capillary electrophoresis on an ABI Prism 3730 Genetic Analyzer (Applied Biosystems). The resultant traces were analyzed using the software GeneMarker 1.70 (SoftGenetics, State College, PA, USA). After population normalization, the peak height from each sample was compared to a synthetic control, which represents the median of all normal samples in each experiment. Peak heights below 0.75 were considered as deletions and values above 1.3 as duplications. Cases with apparent deletions or duplications were confirmed with quantitative PCR. Apparent deletions of a single probe were sequenced to rule out single-base changes within the probe-binding region. Analysis of positive controls (with confirmed 15q11-13 and 22q11.2 deletions and duplications) under the same experimental conditions ensured the reliable detection of copy number gains and loses.

## Results

DNA from 236 unrelated individuals with OCD was screened for 15q11-13 and 22q11.2 microrearrangements by MLPA. No deletions or duplications were identified in any sample in these two loci. Furthermore, no gene dosage abnormalities were detected in the other chromosomal regions screened with the MLPA P064 MR1 kit.

## Discussion

Chromosomal rearrangements reported in individuals with OCD suggest that gene dosage effects could contribute to the determinism of the disorder. To our knowledge, this is the first study to systematically explore CNVs in OCD. As a preliminary study, we screened a sample of OCD patients for CNVs in the 15q11-13 and the 22q11.2 chromosomal regions. These two regions were chosen because patients with 22q11.2 deletion syndrome or PWS have an elevated incidence of obsessive-compulsive symptoms [12,18,20]. We did not detect any microrearrangement in these regions in our sample. If present, the prevalence of these chromosomal anomalies in OCD would be rare, i.e. under 2 × 10^-3 ^(<1/472 chromosomes screened). MLPA is a highly reliable method to detect microrearrangements in the 15q11-q13 and 22q11.2 regions, and has been used with success by our group and others to screen subjects with autism spectrum disorders and mental retardation [22,23,27-29]. Thus, the negative findings in the present OCD sample cannot be ascribed to lack of sensitivity of the method to detect copy number abnormalities.

Despite their heterogeneity, the main clinical characteristics of patients with PWS or 22q11.2 deletion syndrome are relatively well recognized by psychiatrists. The non inclusion of patients with clear dysmorphic features in our study could explain at least in part why we were unable to detect any subjects with such deletions. However, patients with atypical or minimal phenotype (i.e., patients without the congenital heart defects, palate anomalies and distinctive facial features of the 22q11 deletion syndrome or without the characteristic obesity of PWS), would not have been recognized by the psychiatrists and in principle could have been included in the OCD sample. The fact that patients with significant mental retardation were absent from our sample also contributes to explain why we did not identify any subjects with 15q11-q13 or 22q11 microdeletions. Indeed, recent findings have shown that pathogenic CNVs are more frequent among individuals with moderate to severe intellectual disability [30].

Our results also failed to identify any duplication of the 15q11-q13 or 22q11.2 regions. Maternally-derived duplications of chromosome 15q11-q13, involving the region deleted in PWS and Angelman syndrome, confer a high risk of autism spectrum disorder or autistic features, whereas paternal inheritance usually leads to a normal phenotype or mild developmental delay [31,32]. Recent technical progresses have lead to the identification of new chromosomal microrearrangements, including the reciprocal duplications of 22q11.2 deletions [33,34]. 22q11.2 microduplications are characterized by highly variable and subtle phenotypes. The majority of individuals have cognitive deficits including speech delay and developmental delay [33,35]. In addition, 22q11.2 microduplications can be inherited from relatives with no distinctly recognizable phenotype, suggesting reduced penetrance [36]. OCD or obsessive-compulsive symptoms have not been reported in individuals with the 15q11-q13 duplication syndrome or the 22q11.2 duplication syndrome, but given the recent identification of the latter syndrome and the limited number of patients described [33-35,37], further studies are needed.

Several limitations of this study should be taken into account when interpreting its results. It is likely that there is a selection bias in the sample of patients studied. Both in France and in Germany, patients were recruited at OCD outpatient clinics, where individuals with severe developmental disabilities and associated medical conditions are unlikely to come, thus decreasing the possibility of detecting pathogenic CNVs [30]. Second, because the purpose of our study was to detect the large deletions that are typically observed in Prader-Willi and DiGeorge syndromes, we did not screen for small deletions in the 15q11-q13 and 22q11 regions (none of which has been shown to be pathogenic) or for intragenic deletions. An additional limitation of our study is the relatively limited size of our sample. As pathogenic CNVs are rare events, type II errors could explain our inability to detect any rearrangement in the 15q11-q13 and 22q11 regions.

## Conclusions

In conclusion, although our study did not identify 15q11-q13 and 22q11 microdeletions in patients with OCD, further search of CNVs in OCD is warranted using genome-wide approaches in large samples. The recently created OCD International Genetics Consortium will perform such studies with a sufficiently large number of individuals, by pooling DNA from different sites [1,38]. Recent whole genome association studies and CNV analyses using microarray technologies in other neurodevelopmental disorders such as autism and schizophrenia suggest that CNVs are more promising to identify regions of the genome with high probability of harboring candidate genes, than the results of the association study itself [39-42]. The identification of multiple, individually rare structural genomic variants throughout the genome playing a causal role or significantly increasing the risk in neuropsychiatric disorders has resulted in a shift from the 'common disease-common variant' perspective to the 'multiple rare variants' perspective in the conceptualization of these disorders. Similar advances are expected in OCD with the use of genome-wide approaches to identify CNVs conferring an increased risk for the disorder.

## Competing interests

The authors declare that they have no competing interests.

## Authors' contributions

RD was responsible for the ascertainment of the patients from France, was involved in the coordination of the study and drafted the manuscript. DMD was involved in the molecular genetic studies and data analysis and helped to write the manuscript. AG carried out the MLPA experiments and participated in the analysis. WM, RM, HJG, SR, PF and MW recruited the German subjects. PC was involved in the evaluation of the French patients. MCM, ML and MW supervised the clinical evaluation of patients. CB conceived the study and coordinated it, supervised the molecular studies and was responsible for writing the manuscript. All authors read and approved the final manuscript.

## Pre-publication history

The pre-publication history for this paper can be accessed here:

http://www.biomedcentral.com/1471-2350/11/100/prepub
